# RNA analysis based on a small number of manually isolated fixed cells (RNA-snMIFxC) to profile stem cells from human deciduous tooth-derived dental pulp cells

**DOI:** 10.1186/s12575-021-00149-5

**Published:** 2021-06-11

**Authors:** Emi Inada, Issei Saitoh, Naoko Kubota, Yoko Iwase, Yuki Kiyokawa, Hirofumi Noguchi, Youichi Yamasaki, Masahiro Sato

**Affiliations:** 1 Department of Pediatric Dentistry, Graduate School of Medical and Dental Sciences, Kagoshima University, Kagoshima, 890-8544 Japan; 2 Department of Pediatric Dentistry, Asahi University School of Dentistry, Gifu, 501-0296 Japan; 3 Division of Pediatric Dentistry, Graduate School of Medical and Dental Science, Niigata University, Niigata, 951-8514 Japan; 4 Department of Dentistry for the Disabled, Asahi University School of Dentistry, Gifu, 501-0296 Japan; 5 Department of Regenerative Medicine, Graduate School of Medicine, University of the Ryukyus, Okinawa, 903-0215 Japan; 6 Department of Genome Medicine, National Center for Child Health and Development, 2-10-1, Tokyo, 157-8535 Japan; 7 Section of Gene Expression Regulation, Frontier Science Research Center, Kagoshima University, Kagoshima, 890-8544 Japan

**Keywords:** Human deciduous tooth-derived dental pulp cells, Stemness factor, Alkaline phosphatase, Octamer-binding transcription factor 3/4, Manual isolation, Mouthpiece-controlled micropipette, CDNA amplification, RNA analysis, Somatic stem cells, Immuno-staining, Cytochemical staining

## Abstract

**Background:**

Expression of stemness factors, such as octamer-binding transcription factor 3/4 (*OCT3/4*), sex determining region Y-box 2 (*SOX2*), and alkaline phosphatase (*ALP*) in human deciduous tooth-derived dental pulp cells (HDDPCs) can be assessed through fixation and subsequent immuno- or cytochemical staining. Fluorescence-activated cell sorting (FACS), a powerful system to collect cells of interest, is limited by the instrument cost and difficulty in handling. Magnetic-activated cell sorting is inexpensive compared to FACS, but is confined to cells with surface expression of the target molecule. In this study, a simple and inexpensive method was developed for the molecular analysis of immuno- or cytochemically stained cells with intracellular expression of a target molecule, through isolation of a few cells under a dissecting microscope using a mouthpiece-controlled micropipette.

**Results:**

Two or more colored cells (~ 10), after staining with a chromogen such a 3,3′-diaminobenzidine, were successfully segregated from unstained cells. Expression of glyceraldehyde 3-phosphate dehydrogenase, a housekeeping gene, was discernible in all samples, while the expression of stemness genes (such as *OCT3/4*, *SOX2*, and *ALP*) was confined to positively stained cells.

**Conclusion:**

These findings indicate the fidelity of these approaches in profiling cells exhibiting cytoplasmic or nuclear localization of stemness-specific gene products at a small-scale.

## Background

Somatic stem cells (SSCs) are present in various tissues, including bone marrow, and due to their pluripotency and low carcinogenic potential, they have been widely used in regenerative medicine, i.e., repair of injured tissues [1–3]. In the dental field, stem cells isolated from human exfoliated deciduous teeth (SHEDs) have been recognized as SSCs since 2003 [4]. Dental pulp stem cells (DPSCs) (including SHEDs) have the ability for self-renewal, and being multipotent, they differentiate into osteoblasts, chondrocytes, adipocytes, neural cells, and odontoblasts [5, 6]. DPSCs express STRO-1, cluster of differentiation 106 (CD106), and homeobox protein NANOG, all of which are specific markers for mesenchymal stem cells (MSCs) [7]. Furthermore, compared with other DPSCs, SHEDs exhibit a greater rate of proliferation [4, 8, 9], and express the MSC surface markers (CD44, CD73, and CD90), osteoblast markers [alkaline phosphatase (*ALP*), runt-related transcription factor 2 (*RUNX2*), and collagen type I α2 chain (*COL1A2*)], cartilage cell markers [collagen type X α1 Chain (*COL10A1*) and aggrecan (*ACAN*)], adipose cell markers [peroxisome proliferator-activated receptor-gamma2 (*PPAR-γ2*) and lipoprotein lipase (*LPL*)], and neuronal stem cell marker, nestin [10].

Dental pulp derived from a human deciduous tooth, which is naturally replaced by a permanent tooth in children aged 6–12 years, has also been recently considered a useful source of agents used in regenerative medicine because they are thought to contain SSCs in combination with fibroblastic and mesenchymal cells [11, 12]. Unfortunately, little is known about SSCs within the dental pulp, which have been hereinafter called “human deciduous tooth-derived dental pulp cells (HDDPCs).”

The properties of HDDPCs isolated from several donors were previously examined at the immunocytological and molecular levels. It has been demonstrated that 1) expression levels of stemness factors [e.g., octamer-binding transcription factor 3/4 (*OCT3/4*), sex determining region Y-box 2 (*SOX2*), and *ALP* differ among the donors tested, 2) HDDPCs with high ALP activity and enriched with OCT3/4 and SOX2 tend to be easily reprogrammed into induced pluripotent stem cells when they are transfected with vectors carrying Yamanaka’s reprogramming factors, and 3) these ALP-enriched cells have the ability to differentiate into osteogenic or adipose cells when they are induced to differentiate into an osteoblastic or adipogenic lineage [13, 14]. These findings suggested that *ALP*-positive cells (also positive for expression of *OCT3/4* and *SOX2*) in HDDPCs could serve as a useful source in regenerative medicine.

Notably, even within donor HDDPCs, there is heterogeneity with respect to staining upon using stemness factor-specific antibodies [13, 14]. Based on our previous hypothesis, HDDPCs are recognized using anti-OCT3/4 or anti-SOX2 antibodies or by cytochemical staining for ALP activity [14]. Thus, enrichment of these positively stained cells is a prerequisite to characterize HDDPCs. Fluorescence-activated cell sorting (FACS) has been widely employed as a powerful and effective tool for cell enrichment [15]. It is theoretically possible to isolate cells exhibiting cytoplasmic or nuclear localization of molecules, such as, OCT3/4, SOX2, and ALP, using FACS, although cells must be fixed prior to immunoreaction. However, the instruments used for FACS are expensive and typically found only in laboratories, and require special skills for handling. Magnetic-activated cell sorting (MACS) is another commonly used enrichment method [16, 17]. Unfortunately, the use of MACS is confined to cells expressing target molecules on their surface, as this technique employs magnetically labeled antibodies [18].

If mRNA is successfully recovered from fixed and stained cells after exposure to a chromogen such a 3,3′-diaminobenzidine (DAB) and subsequently converted into cDNA prior to amplification, it is theoretically possible to assess the expression of nuclear transcription factors or cytoplasmic factors. This will in turn be helpful for profiling HDDPC-derived cells at the molecular level. In this study, we developed a novel, simple, and inexpensive method to manually isolate fixed and immuno- or cytochemically stained cells under a dissecting microscope using a mouthpiece-controlled micropipette. We call this technology “RNA analysis based on a small number of manually isolated fixed cells (RNA-snMIFxC).” Using this technology, in this study, we aimed to characterize HDDPCs, which can be recognized at the molecular level using anti-OCT3/4 or anti-SOX2 antibodies or by cytochemical staining for ALP activity.

## Results

### Preliminary tests to establish RNA-snMIFxC

**Fig. 1:**
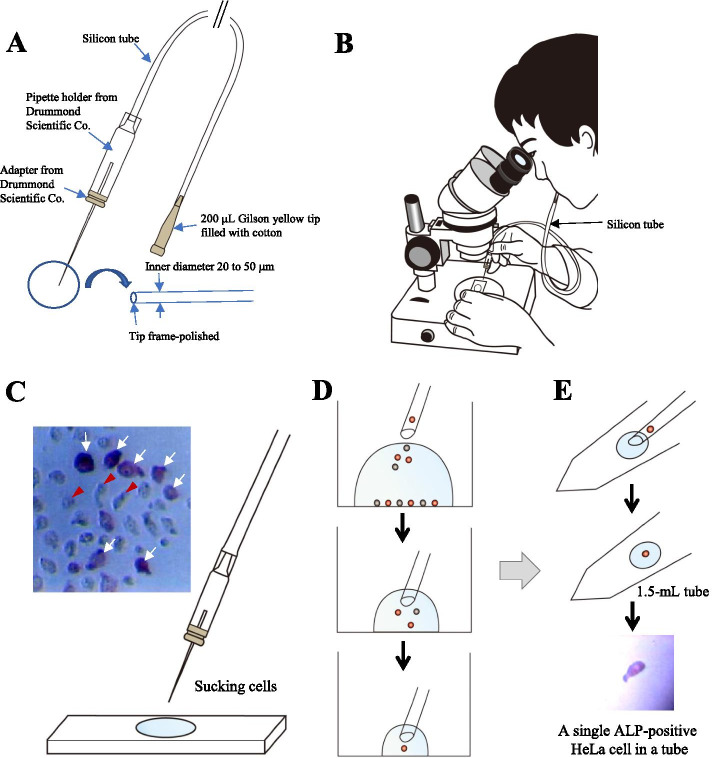
Manual sorting of fixed and immuno- or cytochemically stained cells. (**A**) Micropipette attached to a mouthpiece for manual cell isolation. The inner diameter of micropipette is 20–50 µm and the tip frame is polished. This micropipette is held by a pipette holder, which is connected to a silicon tube and mouthpiece (200-µL tip filled with cotton). (**B**) Picture showing cell isolation by a breath-controlled micropipette under a dissecting microscope. (**C**) Photograph showing HeLa cells after cytochemical staining for alkaline phosphatase (ALP) activity and subsequent cell isolation. As shown in the left upper panel, HeLa cells comprise at least two types of cells, namely extensively ALP-stained cells (arrows) and less stained cells (arrowheads). Twenty microliter of the ALP-stained cell suspension was picked using a 200 µL tip attached to a Gilson Pipette under a dissecting microscope and placed onto a non-adhesive plastic plate. (**D**) Further processing of cells. A few cells (including ALP-positive and negative cells) are collected using a micropipette and placed onto another 1 µL drop. Finally, a single cell is collected into the pipette. (**E**) Placing of cell(s) into a tube. The sorted cell(s) are transferred to a 1 µL drop of Ca^2+^, Mg^2+^-free Dulbecco’s modified phosphate-buffered saline containing 4% fetal bovine serum (DPBS-FBS) in a 1.5 mL microtube. The photograph shown in the bottom panel is a single ALP-positive cell after transfer to a 1 µL drop of DPBS-FBS (in the microtube)

To determine the accuracy of picking single cell(s) using a mouthpiece-controlled micropipette under a dissecting microscope, HeLa cells and HDDPCs, which had been cytochemically stained for ALP activity were used. The process of isolation of single cell (HeLa cell) is shown in Fig. 1A and the cells handling under a dissecting microscope is shown in Fig. 1B. When HeLa cells are cytochemically stained for ALP activity, at least two types of cells are observed [19], namely extensively stained cells (arrows in Fig. 1C) and less stained cells (arrowheads in Fig. 1C). A few cells (including ALP-positive and negative cells) were sucked up with a micropipette, which was controlled by breath through a mouthpiece and placed onto another 1 µL drop (as shown in Fig. 1D). Then, the single cell(s) were picked and transferred to a 1 µL drop in a 1.5 mL microtube (as shown in Fig. 1E). The lower panel of Fig. 1E shows that a single ALP-positive cell has been successfully transferred to a 1 µL drop within a 1.5 mL microtube.

**Fig. 2:**
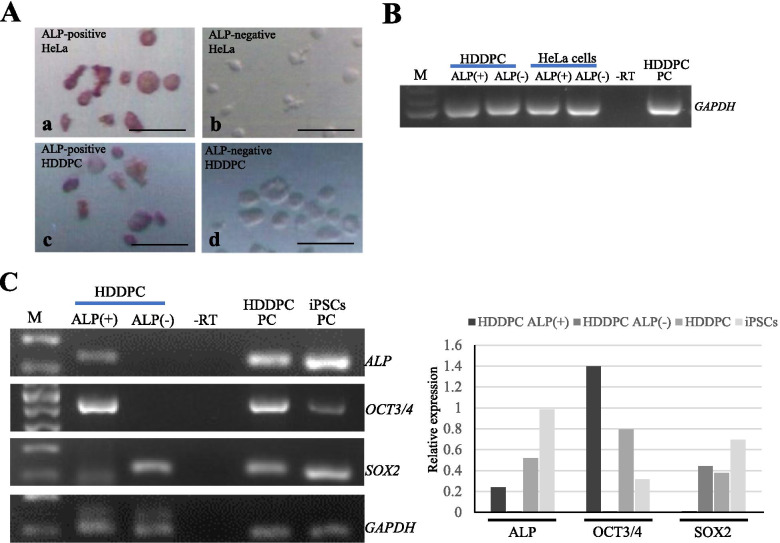
Preliminary tests confirming the accuracy of single-cell RNA-snMIFxC.** (A)** Sorted HeLa and human deciduous tooth-derived dental pulp cells (HDDPCs). There are ~ 10 HeLa cells with high alkaline phosphatase (ALP) activity (a) and those with less ALP activity (b). Similarly, there are ~ 10 HDDPCs with high ALP activity (c) and those with less ALP activity (d). Bar = 10 μm. **(B)** Reverse transcription-polymerase chain reaction (RT-PCR) using whole transcriptome amplification (WTA) products derived from HDDPC-ALP( +), HDDPC-ALP(-), HeLa cells-ALP( +) and HeLa cells-ALP(-) to amplify transcripts of glyceraldehyde 3-phosphate dehydrogenase (*GAPDH*). M, 100 bp ladder; -RT, PCR, performed in the absence of reverse transcription products; HDDPC PC, RT-PCR products using cDNAs derived from unselected HDDPCs; iPSC PC, RT-PCR products using cDNAs derived from unselected HDDPC-derived induced pluripotent cells (iPSCs). **(C)** RT-PCR to check the expression of *ALP,* octamer-binding transcription factor 3/4 (*OCT3/4*) and sex determining region Y-box 2 (*SOX2*) in HDDPC-ALP( +) and HDDPC-ALP(-) using appropriate primer sets. In the right panel, comparison of the expression levels of each transcript between HDDPC-ALP( +) and HDDPC-ALP(-), after normalization is shown. The intensity of each band in the left panel was determined using the ImageJ software. The level of each transcript was normalized to that of *GAPDH* mRNA, and the results obtained are represented as a graph. M, 100 bp ladder; -RT, PCR performed in the absence of reverse transcription products. HDDPC PC, RT-PCR products using cDNAs derived from unselected HDDPCs; iPSC PC, RT-PCR products using cDNAs derived from unselected HDDPC-derived iPSCs

Next, ~ 10 HeLa cells with high and low ALP activity were picked (a vs. b in Fig. 2A). Similar treatment was performed for HDDPCs (c vs. d in Fig. 2A). Starting from these collected cells; mRNA purification, cDNA synthesis, and subsequent cDNA amplification were performed using a whole transcriptome amplification (WTA) kit. When reverse transcription-polymerase chain reaction (RT-PCR) was performed using the WTA products to amplify the transcripts of glyceraldehyde 3-phosphate dehydrogenase (*GAPDH*), a housekeeping gene, a single band of 109 bp with uniform intensity was observed in each lane (Fig. 2B). This suggested that this system can be used for the detection of genes of interest at the mRNA level.

### Gene expression analysis between ALP-positive and -negative HDDPCs

Next, a possible difference in the expression of several stemness factors (e.g., ALP, OCT3/4 and SOX2) between HDDPCs with higher ALP activity (hereinafter referred to as HDDPC-ALP( +)) and those with less ALP activity (referred to as HDDPC-ALP(-)) was examined. Using the WTA products derived from HDDPC-ALP( +) and HDDPC-ALP(-), RT-PCR was performed. Consequently, HDDPC-ALP( +) expressed both *ALP* and *OCT3/4* (Left panel of Fig. 2C). In contrast, HDDPC-ALP(-) expressed *SOX2*, but not *ALP* and *OCT3/4* (Left panel of Fig. 2C). The intensity of each band in the left panel of Fig. 2C was determined using the ImageJ software (National Institutes of Health; http://rsbweb.nih.gov/ij/) and the level of each transcript was normalized to that of *GAPDH* mRNA. The level of *OCT3/4* transcript in HDDPC-ALP( +) appeared to be higher than that in unselected HDDPCs and iPSCs (right panel of Fig. 2C). This suggested that OCT3/4 expression was relatively high in HDDPC. However, ALP expression was not as high, because the level of *ALP* transcripts in HDDPC-ALP( +) was lower than that in unselected HDDPCs and iPSCs (right panel of Fig. 2C). These results also suggested that ALP was a useful marker for segregating cell populations expressing different stemness factors.

### Gene expression analysis between OCT3/4-positive and -negative HDDPCs or between SOX2-positive and -negative HDDPCs

**Fig. 3:**
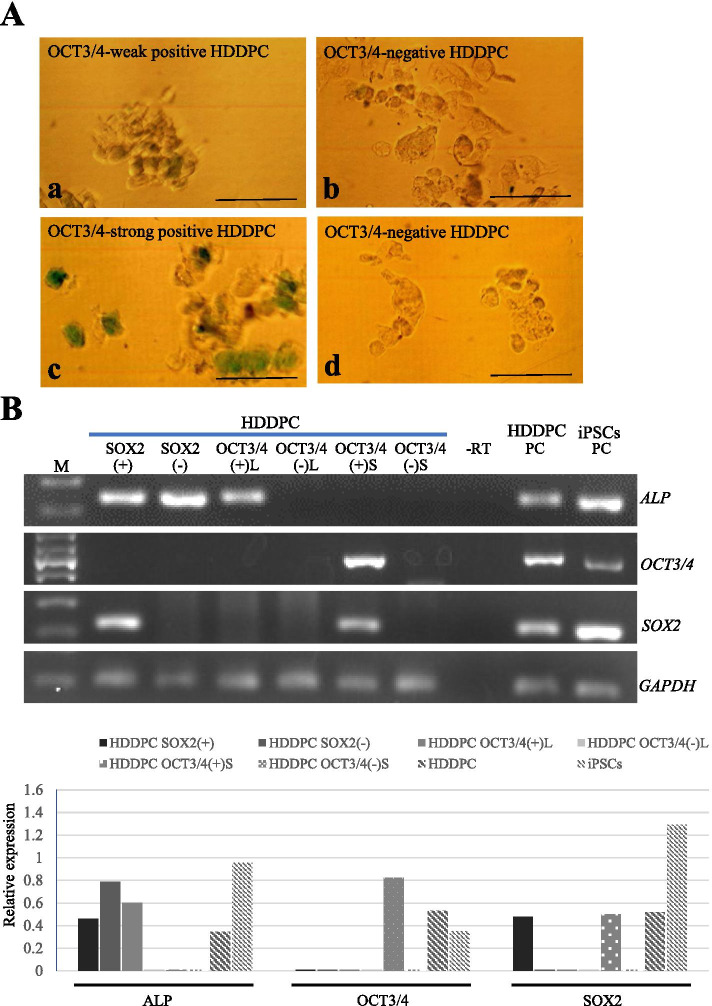
RT-PCR analysis of HDDPCs collected through RNA-snMIFxC.** (A)** HDDPCs sorted. There are ~ 10 HDDPCs with weakly positive octamer-binding transcription factor 3/4 (OCT3/4) activity (a, defined as HDDPC-OCT3/4( +)L) and those with negative OCT3/4 activity (b, defined as HDDPC-OCT3/4(-)L). Similarly, there are ~ 10 HDDPCs with strongly positive OCT3/4 activity (c, defined as HDDPC-OCT3/4( +)S) and those with negative OCT3/4 activity (d, defined as HDDPC-OCT3/4(-)S). Bar = 100 μm. **(B)** RT-PCR analysis of HDDPC-sex determining region Y-box 2 (SOX2)( +), HDDPC-SOX2(-), HDDPC-OCT3/4( +)L, HDDPC-OCT3/4(-)L, HDDPC-OCT3/4( +)S and HDDPC-OCT3/4(-)S using primer sets for alkaline phosphatase (*ALP*)*, OCT3/4*, *SOX2* and glyceraldehyde 3-phosphate dehydrogenase (*GAPDH*). In the lower panel, comparison of the expression levels of each transcript between HDDPC-SOX2( +) and HDDPC-SOX2(-), HDDPC-OCT3/4( +)L and HDDPC-OCT3/4(-)L or HDDPC-OCT3/4( +)S and HDDPC-OCT3/4(-)S, after normalization, is shown. The intensity of each band in the left panel was determined using The ImageJ software. The level of each transcript was normalized to that of *GAPDH* mRNA, and the results obtained are represented as a graph. M, 100 bp ladder; -RT, PCR performed in the absence of reverse transcription products; HDDPC PC, RT-PCR products using cDNAs derived from unselected HDDPCs; iPSC PC, RT-PCR products using cDNAs derived from unselected HDDPC-derived induced pluripotent cells (iPSCs)

In a previous study, HDDPCs were successfully immuno-stained with antibodies raised against OCT3/4 or SOX2 [13, 14]. To examine the possible difference in gene expression between OCT3/4-positive and -negative HDDPCs (or between SOX2-positive and -negative HDDPCs), paraformaldehyde (PFA)-fixed HDDPCs were subjected to permeabilization before reaction with anti-OCT3/4 or anti-SOX2 antibodies, and then probed using a peroxidase-conjugated secondary antibody. A positive reaction was visualized (green color) by incubating cells in a solution containing HistoGreen, a new alternative to 3,3′-diaminobenzidine-tetrahydrochloride-dihydratediaminobenzidine (DAB). A total of ~ 10 cells each, positively and negatively stained with the antibody, were picked (Fig. 3A). In the case of cells stained with the anti-OCT3/4 antibody, cells staining weakly (Fig. 3A a) or strongly (Fig. 3A c) were separated as different groups. In the former group, OCT3/4-positive cells were termed HDDPC-OCT3/4( +)L, and OCT3/4-negative cells were termed HDDPC-OCT3/4(-)L, because it took a relatively longer time (2–3 min) after reaction with HistoGreen to collect HDDPC-OCT3/4( +)L. Similarly, in the latter group, OCT3/4-positive cells were defined as HDDPC-OCT3/4( +)S, and OCT3/4-negative cells were defined as HDDPC-OCT3/4(-)S, because it took a relatively shorter time (less than 1 min) after reaction with HistoGreen to collect HDDPC-OCT3/4( +)S. Thus, HDDPC-OCT3/4( +)S appeared to exhibit stronger OCT3/4 expression than HDDPC-OCT3/4( +)L. In the case of cells stained with the anti-SOX2 antibody, SOX2-positive cells were defined as HDDPC-SOX2( +) and SOX2-negative cells were defined as HDDPC-SOX2(-) (data not shown).

Next, these collected cells were subjected to mRNA purification, cDNA synthesis, and subsequent cDNA amplification using a WTA kit. In the upper panel of Fig. 3B, the results of RT-PCR, when the WTA products were used as templates, are shown. HDDPC-OCT3/4( +)S expressed both *OCT3/4* and *SOX2*, but failed to express *ALP*. In contrast, HDDPC-OCT3/4( +)L expressed *ALP*, but did not express *OCT3/4* and *SOX2*. Neither HDDPC-OCT3/4(-)L nor HDDPC-OCT3/4(-)S were negative for the expression of *ALP*, *OCT3/4*, and *SOX2*. HDDPC-SOX2( +), but not HDDPC-SOX2(-), expressed *SOX2*. In contrast, both types of cells expressed *ALP*, but not *OCT3/4*. The level of each transcript was normalized to that of *GAPDH* mRNA. The level of *OCT3/4* transcript in HDDPC-OCT3/4( +)S appeared to be higher than that in unselected HDDPCs and iPSCs (lower panel of Fig. 3B), which was in contrast with the findings represented in the right panel of Fig. 2C. These findings suggested that there were various types of cells among the HDDPC population.

### RNA-snMIFxC enabled the detection of stemness factor expression even when mRNA purification started from only two cells

**Fig. 4:**
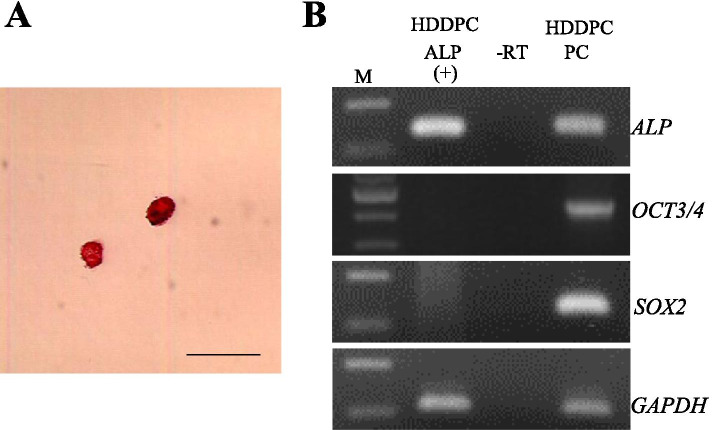
RT-PCR analysis of two ALP-positive HDDPCs collected through RNA-snMIFxC.** (A)** Two alkaline phosphatase (ALP)-positive HDDPCs sorted. Bar = 100 μm. **(B)** RT-PCR analysis of HDDPC-ALP( +) using primer sets for *ALP,* octamer-binding transcription factor 3/4 (*OCT3/4*)*,* sex determining region Y-box 2 (*SOX2*) and glyceraldehyde 3-phosphate dehydrogenase (*GAPDH*). M, 100 bp ladder; -RT, PCR performed in the absence of reverse transcription products. HDDPC PC, RT-PCR products using cDNAs derived from unselected HDDPCs

In previous experiments, ~ 10 cells were used to detect stemness factor expression using the RNA-snMIFxC system. This confirmed that gene expression profiling was possible when the analysis started from that number of cells. In this test, it was determined whether RNA-snMIFxC was also effective when analysis started from only two cells. Two ALP-positive HDDPCs were picked, as shown in Fig. 4A. These cells were then subjected to WTA. RT-PCR of these WTA products demonstrated that expression of both the *ALP* and *GAPDH* genes was discernible (Fig. 4B). Notably, in this case, the samples used were negative for expression of both *OCT3/4* and *SOX2*. From these experiments, it was found that RNA-snMIFxC was effective for detecting gene expression in very few cells.

## Discussion

There are two major technical issues when the analysis is conducted using a small number of cells. One is the difficulty in isolating target cells from a heterogeneous cell population under an environment while avoiding any possible contamination [20, 21], and the other is to amplify a trace amount of mRNA without noise or bias amplification. For the former, several methods, including FACS, MACS, microfluidics, optical tweezers, and micromanipulation are now available [21–23]. For example, microfluidics has an advantage of enabling the isolation of a small number of cells, but is often costly [24]. Optical tweezers employ lasers instead of a micropipette to pick cells, and enable more accurate cell isolation when compared to micropipettes; unfortunately, this system is costly and is used in only a limited number of laboratories [23]. Micromanipulation-based cell isolation employs a micropipette that is connected to a micromanipulator, and is always done via viewing cells under a microscope; in this context, this micromanipulation-based cell isolation technique appears to be among the low-throughput systems [25]. However, skilled personnel are required to perform the isolation. In this study, we developed a manual isolation approach using a micropipette as a simple and convenient alternative to these methods. Unlike the micromanipulation-based cell isolation, cell collection was controlled by breath through a silicone tube attached to a mouthpiece. Notably, this approach has widely and long been employed for studying mammalian embryogenesis as a low-cost preimplantation embryo handling tool [26]. To our knowledge, this is the first report on the employment of manual cell isolation using a breath-controlled micropipette.

Our system is based on cell isolation under a dissecting microscope, as shown in Fig. 1B. In this case, it is convenient to pick colored cells, which have been immuno- or cytochemically stained after fixation of cells. Fixed cells are often used for the purpose of cell isolation [27–29]. Among numerous fixatives, a weak formaldehyde solution has been routinely used as one of the typical fixatives. Formaldehyde usually cross-links nucleic acids (NAs) to proteins and causes chemical modifications of RNA, DNA, and protein. Thus, the integrity of NAs is compromised, limiting the efficiency of isolation, detection, and accurate quantitation [30]. However, previous reports demonstrated that quantitative analysis using RT-PCR is possible when formalin-fixed paraffin embedded tissue is used as a starting material [31, 32]. It is also reported that the quantity of RT-PCR products did not differ significantly before and after fixation, although several factors (e.g., amplicon size and the time for fixation) are thought to affect quality in RT-PCR-based assays [30, 33, 34]. In this study, we found that cells that have been fixed, and subsequently immuno- or cytochemically stained are suitable for use in molecular analysis.

**Fig. 5:**
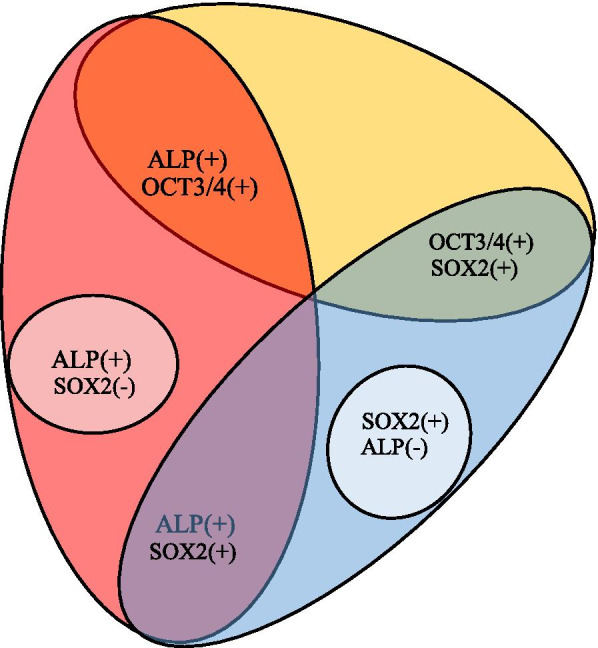
Pattern diagram showing various types of HDDPCs with respect to the expression of the stemness factors. Among OCT3/4-positive HDDPCS, some cells express *SOX2*, but other cells do not. ALP-positive cells express both *ALP* and *OCT3/4* or *OCT3/4* alone, but ALP-negative cells fail to express both. OCT3/4-positive cells express *ALP* or *SOX2*. However, there are no cells expressing the three factors simultaneously. Furthermore, some HDDPCs expressed *SOX2* even in the absence of *ALP* expression

Notably, this appears to be the first analysis of stemness factors, as exemplified by *OCT3/4*, *SOX2*, and *ALP*, using a small number of isolated HDDPCs. In our previous study using an immunocytochemical approach, it has been noted that there are markers that are expressed in HDDPCs, but they exhibit a mosaic distribution pattern [14]. To examine whether or not the expression of these markers overlapped with each other, we manually isolated a few fixed, and immuno- or cytochemically stained HDDPCs for RT-PCR. Consequently, HDDPC-ALP( +) expressed both *ALP* and *OCT3/4*, but HDDPC-ALP(-) expressed only *SOX2* (see Fig. 2C). These results indicated a good correlation between the results of immuno- or cytochemical staining and the results obtained through RNA-snMIFxC, and suggested that expression of stemness factors overlapped in some cells, but not in others. A similar tendency was also seen when HDDPCs were immuno-stained with anti-OCT3/4. OCT3/4-positive cells (designated as HDDPC-OCT3/4( +)S) expressed the *OCT3/4* mRNA, as detected by RT-PCR, but the other cells (designated as HDDPC-OCT3/4( +)L) did not (see Fig. 3B). This discrepancy may be due to the different staining times after reaction with HistoGreen. HDDPC-OCT3/4( +)S were removed from the HistoGreen-containing solution at an earlier time (i.e., within 1 min after reaction with HistoGreen) than HDDPC-OCT3/4( +)L (i.e., 2–3 min after reaction with HistoGreen), suggesting that the former cells may more strongly express *OCT3/4* than the latter cells. These results also suggested that HDDPCs consisted of heterogeneous cells [as shown by OCT3/4( +)/ALP( +), OCT3/4( +)/SOX2( +), ALP( +) or ALP(-)/SOX2( +)], as summarized in Fig. 5.

Furthermore, we found that the level of *OCT3/4* transcripts in HDDPC-ALP( +) appeared to be higher than that in unselected HDDPCs and iPSCs (see right panel of Fig. 2C). A similar pattern was also observed when comparing *OCT3/4* expression between HDDPC-OCT3/4( +)S and HDDPC-OCT3/4(-)S (lower panel of Fig. 3B). This was unexpected, because *OCT3/4* is one of the molecules abundantly expressed in iPSCs [35]. Notably, it has been reported that expression of *OCT4* in somatic cells is very low, therefore suggesting that *OCT3/4* lacks a functional role in somatic cells [36, 37]. Isolation of viable *OCT3/4*-positive HDDPCs is necessary for elucidating the role of OCT3/4 in somatic cells. In this context, studies on the role of OCT3/4 in somatic cells are currently underway.

## Conclusion

cDNAs using WTA, starting from a few (~ 10) fixed and immuno- or cytochemically stained cells that had been manually isolated by a mouthpiece-controlled micropipette were successfully amplified. RT-PCR using specific primers revealed that HDDPCs showing a positive reaction after staining also expressed target mRNA, suggesting the accuracy of RNA-snMIFxC. Furthermore, there were various cell populations among HDDPCs, as exemplified by OCT3/4( +)/ALP( +) and OCT3/4( +)/SOX2( +) cells. As *OCT3/4*, *SOX2*, and *ALP* are well known stemness factors highly expressed in undifferentiated pluripotent SSCs, it is highly likely that HDDPCs might contain pluripotent stem cells. Future studies using these SSCs from HDDPCs would provide more insights on the role of these cells in dentinogenesis as well as the possible use of these cells in regenerative medicine.

## Materials & Methods

### Cells

Human deciduous teeth were extracted from a healthy individual because of replacement failure in our department under approved guidelines set by the ethical committee for the use and experimentation of the Kagoshima University Graduate School of Medical and Dental Science (permission no. 27–11; valid from May 29, 2015 to March 31, 2020). Informed consent was obtained from the subjects or their parents prior to their entry into the study. HDDPCs were removed from deciduous teeth and digested in a solution of 3 mg/mL collagenase type I (#17,100–017; Invitrogen, Carlsbad, CA, USA) and 4 mg/mL dispase (#410,810,077; Roche Applied Science, Basel, Switzerland) for 30–60 min at 37 °C. The isolated pulp cells were seeded onto 60 mm gelatin-coated dishes (#4010–020; Iwaki Glass Co. Ltd., Tokyo, Japan) containing Dulbecco’s modified Eagle’s medium (DMEM; #11,995–081; Invitrogen) supplemented with 20% heat-inactivated fetal bovine serum (FBS; #SFMB30-2239; Equitech Bio Inc., Kerrville, TX, USA), 50 U/mL penicillin, and 50 mg/mL streptomycin (#15,140–122; Invitrogen) (DMEM/20% FBS) and were cultured at 37 °C in an atmosphere of 5% CO_2_. Media were changed every 3 days. The MT line [14] showing ALP activity was used for experiments at approximately passage six.

To verify the validity of the developed method, HeLa cells [#CRL-1469™; American Type Culture Collection (ATCC), Manassas, VA, USA], the gene expression profile of which has previously been investigated [19]. were used. Cells were grown in DMEM supplemented with 10% FBS at 37 °C in an atmosphere of 5% CO_2_.

The HDDPC-derived iPSCs generated in our laboratory[38] were cultured on mitomycin C (MMC) (#M4287; Sigma-Aldrich, St. Louis, MO, USA)-treated MEFs in a 60 mm gelatin-coated dish (#4010–020; Iwaki Glass Co. Ltd., Tokyo, Japan); these cells were cultured in human ES cell culture medium iPSellon (#007,001; Cardio, Kobe, Japan), supplemented with 5 ng/mL recombinant human bFGF (#064–04,541; Wako Pure Chemical Industries, Ltd., Osaka, Japan) and 0.01 µg/mL recombinant human LIF (#129–05,601; Wako Pure Chemical Industries, Ltd.). The medium was changed every day by replacing half of the medium with fresh medium. Passaging was performed using trypsinization on the fifth day after cell seeding.

### Manual sorting of fixed and cytochemically stained cells

Cells were cultivated in 35 mm gelatin-coated dishes (#4000–020; Iwaki Glass Co. Ltd.) until 80%–90% confluence. Cells were detached using 0.25% trypsin in Ca^2+^, Mg^2+^-free Dulbecco’s modified phosphate-buffered saline (DPBS) and transferred to a 1.5 mL microtube. After a brief precipitation of cell pellets, cells were fixed by adding 200 µL of 4% PFA (#161–20,141; Wako Pure Chemicals Inc., Osaka, Japan) in DPBS, left for 5 min at room temperature (approximately 25 °C), and then washed with DPBS.

Prior to manual sorting of fixed cells, a hand-made micropipette attached to a mouthpiece was used to isolate single cells. This type of pipette has been frequently used for handling preimplantation mouse embryos in vitro [26]. The micropipette was manually prepared by pulling a glass pipette (#2–000-050; Drummond Scientific Co. Broomall, PA, USA) in a flame.

The cytochemical assay for detecting ALP activity was performed using a Leukocyte Alkaline Phosphatase Kit (#ALP-TK1; Sigma-Aldrich, St. Louis, MA, USA). ALP-positive cells were visualized as red brown products by staining with α-naphthol as a substrate for ALP and binding with a diazonium salt. One day later, α-naphthol was replaced with 200 µL of DPBS containing 4% FBS (referred to as DPBS-FBS). Twenty microliters of the ALP-stained cell suspension was placed onto a non-adhesive plastic plate, and a single cell or more cells (~ 10) for each ALP-positive or ALP-negative group were collected using the micropipette (attached to a mouthpiece) under observation with a dissecting microscope. The sorted cell(s) were transferred to a 1 µL drop of DPBS-FBS in a 1.5 mL microtube and stored at -20 °C until analysis.

### Manual sorting of fixed and immunocytochemically stained cells

Cells cultured in a dish were harvested and fixed, as described in Sect. 4.2. After washing with DPBS, cells were permeabilized via incubation in 0.1% Triton X- 100 (#T8787; Sigma-Aldrich) for 3 min at room temperature. After washing with DPBS containing 1% normal goat serum (NGS) (Invitrogen) (referred to as DPBS-NGS), cells were blocked via incubation in 20% AquaBlock tm/EIA/WB (#PP82; East Coast Biologics, Inc., North Berwick, USA) for 30 min at 4 °C. After washing with DPBS-NGS, cells were stained with primary antibodies against OCT3/4 (#sc-9081; clone H-134, 1:200; Santa Cruz Biotechnology, Dallas, TX, USA) or SOX2 (#SAB2701974; 1:200; Sigma-Aldrich) overnight at 4 °C. After washing with DPBS-NGS, cells were reacted with the secondary antibody, goat anti-rabbit IgG H&L conjugated with horseradish peroxidase (#ab205718; 1:200; Abcam, Cambridge, UK) for 2 h at 4 °C. After washing with DPBS-NGS, cells were reacted with HistoGreen (#E109; Eurobio Ingen, Les Ulis, France) for approximately 0.5–3 min at room temperature. Since HistoGreen is a substrate chromogen adapted for peroxidase-based immunohistochemical staining, care is taken when the reaction is excessively processed. If this occurred, the reaction was immediately stopped by moving the reactive mixture to a water drop using a mouthpiece-controlled micropipette. When cells properly reacted to HistoGreen, they were stained green. The HistoGreen-positive or -negative cells were collected, as described in Sect. 4.2.

### cDNA synthesis and amplification

The collected single cell(s) were next subjected to WTA using the NEBNext Single Cell/Low Input cDNA Synthesis & Amplification Kit (NEB #E6421; New England Biolabs Japan Inc., Tokyo, Japan). The manufacturer’s protocol was followed from mRNA purification to amplification of cDNA.

First, the fixed cells were dispensed in cell lysis solution containing 0.5 µL of cell lysis buffer, 0.25 µL of murine RNase inhibitor and 4.25 µL of nuclease-free water, and incubated at room temperature for 5 min. To anneal the resulting mRNA with primers, 1 μL of Single Cell RT Primer Mix and 3 µL of nuclease-free water were added to the cDNA (9 μL) in a 0.5 mL microtube (#PCR-05-C-J; Axygen Inc., Corning, NY, USA) and incubated at 70 °C for 5 min in a thermal cycler (Gene Atlas 482; Astec, Fukuoka, Japan). Next, for reverse transcription and template switching, 5 µL of Single Cell RT Buffer, 1 µL of Template Switching Oligo, 2 µL of Single Cell RT Enzyme Mix, and 3 µL of nuclease-free water were added to the 0.5 mL microtube containing annealed mRNA and incubated at 42 °C for 90 min, and then at 70 °C for 10 min. Finally, cDNA amplification using PCR was performed in a total volume of 80 µL solution containing 50 µL of Single Cell cDNA PCR Master Mix, 2 µL of Single Cell cDNA PCR Primer, and 28 µL of nuclease-free water. PCR was performed with 20 cycles of 98 °C for 10 s, 62 °C for 15 s, and 72 °C for 3 min using a Gene Atlas 482 thermal cycler.

### RT-PCR analysis

For detection of expression of endogenous *OCT-3/4*, *SOX2*, *ALP*, and *GAPDH* mRNA by RT-PCR, the undiluted cDNA samples (2 μL) were amplified in the first PCR in a total volume of 20 μL using the AmpliTaq Gold® 360 Master Mix (#4,398,881; Applied Biosystems, Foster City, CA, USA) with primer sets listed in Table 1. In this case, cDNA obtained from the usual reverse transcription using HDDPC-derived RNA (~ 0.5 μg) was used as a positive control. As a negative control, a no-template control (designated -RT) was included for each reaction. PCR was performed with 35 cycles of denaturation at 95 °C for 30 s, annealing at 52 °C for 30 s, and extension at 72 °C for 30 s using a Gene Atlas thermal cycler. The resulting products (1 μL) were then subjected to nested PCR in a total volume of 20 μL using the AmpliTaq Gold® 360 Master Mix with primer sets listed in Table 1. The PCR conditions were the same as mentioned above. The products (3 µL) were then analyzed using 2% agarose gel electrophoresis and visualized after staining with ethidium bromide.

**Table 1:** Primer sets used for PCR analysis

Target gene	Primer sequence (5’-3’)	Amplicon size (bp)	Sequence ID
Used for first PCR			
*ALP*	Forward: TGG CCC CCA TGC TGA GTG ACA C Reverse: TGG CGC AGG GGC ACA GCA GAC	160	NM_000478.4
*OCT3/4*	Forward: ATT TCA CCA GGC CCC CGG CT Reverse: GCT GAT CTG CTG CAG TGT GGG	434	NM_002701.4
*SOX2*	Forward: TAA ATA CCG GCC CCG GCG GA Reverse: AGG TCC ATT CCC CCG CCC TC	131	NM_003106.2
*GAPDH*	Forward: TCC AAA ATC AAG TGG GGC GAT GCT GGC GCT GAG TAC Reverse: GTT GTC ATA CTT CTC ATG GTT CAC ACC CAT GAC GAA	180	NM_002046
Used for nested PCR			
*ALP*	Forward: CTA AGT GAC ACA GAC AAG AAG Reverse: GGC ACA GCA GAC TGC GCC TGG	140	NM_000478.4
*OCT3/4*	Forward: GGG ACA CCT GGC TTC GGA TTT CGC Reverse: TCT TTC TGC AGA GCT TTG ATG TCC TG	428	NM_002701.4
*SOX2*	Forward: AGC TAC AGC ATG ATG CAG GAC CAG C Reverse: GTC ATG GAG TTG TAC TGC AGG GCG	125	NM_003106.2
*GAPDH*	Forward: GTC GTG GAG TCC ACT GGC GTC Reverse: ACA TGG GGG CAT CAG CAG AGG	109	NM_002046

Abbreviations: *ALP*, alkaline phosphatase; *OCT3/4*, octamer-binding transcription factor 3/4; *SOX2*, sex determining region Y-box 2; *GAPDH*, glyceraldehyde 3-phosphate dehydrogenase

The intensity of each band in the PCR images was determined using the ImageJ software. The densitometric data of each transcript were normalized with those of the internal control, i.e., *GAPDH* mRNA, and the results were expressed as graphs, in accordance with the method proposed by Chapman et al. [39].

## Data Availability

Not applicable.

## Abbreviations

SSCs: Somatic stem cells
SHED: Stem cells isolated from human exfoliated deciduous teeth
DPSCs: Dental pulp stem cells
CD106: Cluster of differentiation 106
ALP: Alkaline phosphatase
RUNX2: Runt-related transcription factor 2
COL1A2: Collagen type I α2 chain
COL10A1: Collagen type X α1 Chain
ACAN: Aggrecan
PPAR-γ2: Peroxisome proliferator-activated receptor-gamma2
LPL: Lipoprotein lipase
HDDPCs: Human deciduous tooth-derived dental pulp cells
OCT3/4: Octamer-binding transcription factor-3/4
SOX2: Sex determining region Y-box 2
ALP: Alkaline phosphatase
FACS: Fluorescence-activated cell sorter
MACS: Magnetic-activated cell sorting
DAB: 3,3'-Diaminobenzidine-tetrahydrochloride-dihydratediaminobenzidine
RT-PCR: Reverse transcription-polymerase chain reaction
WTA: Whole transcriptome amplification
GAPDH: Glyceraldehyde-3-phosphate dehydrogenase
PFA: Paraformaldehyde
NAs: Nucleic acids
DMEM: Dulbecco’s modified Eagle’s medium
FBS: Fetal bovine serum
DPBS: Dulbecco’s modified Ca^2^^+^, Mg^2^^+^-free phosphate-buffered saline
NGS: Normal goat serum
